# Assessing the Application of a Genomic Network Analysis in Population Ecology: Inferring Patterns of Dispersal and Geographic Structure in the Emerging Pathogen, *Coccidioides*

**DOI:** 10.1002/ece3.73452

**Published:** 2026-04-07

**Authors:** Cari D. Lewis, Morgan E. Gorris, Kimberly A. Kaufeld, Andrew W. Bartlow

**Affiliations:** ^1^ Los Alamos National Laboratory Los Alamos New Mexico USA

**Keywords:** *Coccidioides*, disease ecology, network analysis, population ecology, Valley fever

## Abstract

A challenge in population ecology studies is identifying how to best group individuals into populations, especially when individual origin is unknown. Machine learning has improved upon traditional methods of identifying population structure and is more efficient at handling large, complex datasets. We demonstrate the applicability of a machine learning method to identify hierarchical population structure in an emerging pathogen, *Coccidioides* spp., the causative agent of Valley fever. We compared the network clusters to structure identified by traditional tools as a validation of the network performance. We used publicly available whole‐genome data for 48 *C. immitis* and 102 *C. posadasii*, resulting in 168,211 genome‐wide SNPs among the two species. The network analysis grouped samples into populations comparable to the literature for these species but also identified fine‐scale geographic structure and travel‐associated cases not reported thus far. Exploring different resolutions in the network made it easy to identify unique genotypes specific to California and possibly Nevada, as well as Phoenix‐ and Tucson‐acquired infections in non‐endemic areas, regardless of reported travel history. The present study provides a promising example of how a ML‐based network analysis can improve our ability to understand pathogen ecology, group cases into populations and infer travel‐associated infections.

## Graphical abstract

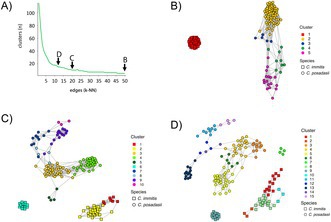

## 1 Introduction

Understanding the ecology of a disease system has become more complex as globalization creates new transmission pathways and introduces pathogens outside of their historical ranges (Baker et al. 2022; Oppong 2020; Frenk et al. 2011). The global health threat posed by complex host‐pathogen‐environment relationships combined with potential long‐distance dispersal and transmission underscores the need for continued disease monitoring. The ability to quickly and efficiently identify infection source location and at‐risk populations is pertinent to biosurveillance and pandemic prevention. A challenge in population studies is identifying how to best group individuals into populations, especially when individual origin is unknown. As became apparent with the emergence of SARS‐CoV‐2, using genomic tools can provide valuable information on pathogen origin and patterns of transmission (Tegally et al. 2023; Biek et al. 2007). The concerted response to the pandemic resulted in numerous computational tools to quickly and efficiently analyze new SARS‐CoV‐2 genomes (Hufsky et al. 2021). However, assessing genomic patterns in emerging infectious pathogens (and non‐model organisms) requires optimization of existing tools. This can be time consuming since traditional population genetics tools like STRUCTURE (Pritchard et al. 2000) require assigning populations a priori, except source populations may be unknown for emerging pathogenic agents. While tools like STRUCTURE, ADMIXTURE (Alexander et al. 2009), and DAPC (Jombart et al. 2010) can infer population source, they are limited to the broadest pattern of structure and are not inherent to handling substructure. More recent approaches like fastSTRUCTURE (Raj et al. 2014) and fineSTRUCTURE (Lawson et al. 2012) are less computationally expensive and better estimate substructure, respectively. Different tools are intended to harness genomic variation to identify evolutionary processes such as historic patterns of gene flow, barriers to migration, and current population structure. Another approach for identifying population structure and patterns of dispersal among increasingly complex datasets worth further exploring is machine learning.

As whole‐genome sequencing has become more accessible for non‐model organisms, the amount of genomic data available for pathogens under surveillance has grown and calls for efficient methods to analyze such data. Various machine learning (ML) techniques have been used to identify disease phenotypes from SNP data associated with hepatitis B seroclearance (Silva et al. 2022), schizophrenia (Aguiar‐Pulido et al. 2010), and other complex polygenic diseases (Elgart et al. 2022). Unlike ML methods that require a training dataset, unsupervised genomic networks are data‐driven ML tools that identify patterns among samples in a dataset and are a promising option for genomic analyses (Gustani‐Buss et al. 2025; Chan et al. 2022; Tay et al. 2022; Witte et al. 2021; Steinig et al. 2019). An unsupervised network can identify multiple patterns from broad‐ to fine‐scale structure in a hierarchical fashion as well as infer likely source populations from dense, whole‐genome SNP datasets (Greenbaum et al. 2019; Neuditschko et al. 2012). These methods have the potential to aid in biosurveillance efforts by identifying pathogen populations from a mixture of isolation sources as well as unknown cases; however, a thorough comparison of methods is lacking.

Our pathogen/disease study system is coccidioidomycosis, or Valley fever—an emerging infectious fungal disease of concern in the Americas. Each year the United States reports 10,000–20,000 cases of Valley fever (CDC 2024), though true disease burden estimates may be up to 18 times higher (Williams et al. 2025). Valley Fever is caused by two dimorphic fungi, *Coccidioides immitis* and *C. posadasii* that have a saprophytic or pathogenic life cycle. Over the past two decades, the incidence of reported Valley fever cases has increased and is linked to substantial morbidity within Arizona, California, and Washington state (McCotter et al. 2019). However, cases have been reported across a broad geographic range in the U.S., likely in association with travel (Monroy‐Nieto et al. 2023). Incomplete metadata and global travel can complicate infection tracking and prevention efforts. Additionally, Valley fever cases are linked to climate variables (Porter et al. 2024; Head et al. 2022) and are expected to increase as its range expands in response to global climate change (Gorris et al. 2019), so understanding the relationships between *Coccidioides* population structure and patterns of infection will help disease mitigation efforts.

Here, we use publicly available whole genome data for both *C. immitis* and *C. posadasii* to assess the applicability of a genetic network analysis pipeline to (1) identify hierarchical population structure between and among the two species, (2) infer population assignments, and (3) assess patterns of travel‐acquired infections. Harnessing genetic diversity and differentiation at multiple geographic scales can provide historical context of gene flow, indicate isolated populations with limited gene flow, and infer contemporary structure from the most informative genomic variants. Improving our ability to identify infection source from cases in non‐endemic areas and unknown travel history can aid in mapping endemic regions for *Coccidioides* and better inform mitigation efforts.

## 2 Materials and Methods

### 2.1 Whole‐Genome Sequence (WGS) Data

We downloaded publicly available WGS data for *C. posadasii* (*n* = 108) and *C. immitis* (*n* = 79) from NCBI (Table S1) using *sratoolkit* v3.0.0 (SRA Toolkit Development Team 2022). Raw sequence data quality was assessed with *fastQC* v0.12.1 (Andrews 2010) and trimmed with default parameters using *Trimmomatic* v0.33 (Bolger et al. 2014). Post‐trimmed *fastQC* statistics are provided in Table S1 for each sample. We aligned the cleaned sequences to the chromosome‐level reference genome of *C. posadasii* (NCBI Assembly GCF_018416015.2, Teixiera et al. 2022) for chromosomes 1–5 using *bwa* v0.7.17 (Li and Durbin 2009). Post‐trimmed alignment metrics were assessed using tools from *GATK* v4.2.0.0 (McKenna et al. 2010) and are reported in Table S1. The reference‐aligned files were converted into a single *mpileup* using *SAMtools* v1.16.1 (Li et al. 2009) and genome‐wide SNPs called using *VarScan* v2.4.6 *mpileup2cns* (Kolboldt et al. 2009). *VarScan* called SNPs that were present at a minimum of 5× coverage (‐‐min‐coverage 5), a minimum quality score of 30 (‐‐min‐avg‐qual 30), and minor allele frequency of 0.01 (‐‐min‐var‐freq 0.01). We also used *SAMtools* to find the percent of genome coverage with a minimum of 5× sequencing depth for each isolate (Table S1). Since *VarScan* defaults to diploid calls, heterozygous loci were excluded and homozygous alleles were collapsed to a haploid dataset using in‐house scripts. We applied additional SNP filters using *VCFtools* v0.1.17 (Danecek et al. 2011) where only biallelic SNPs were retained (‐‐remove‐indels ‐‐min‐alleles 2 ‐‐max‐alleles 2) that were present in at least 90% of individuals (‐‐max‐missing 0.9) and the SNPs were thinned within a 100 bp window to reduce signatures of linkage (‐‐thin 100). The initial dataset contained 169,101 genome‐wide SNPs present across both species (*n* = 183), but a kinship analysis in *VCFtools* (−‐relatedness) identified pairs of individuals that share > 99.9% of SNPs (Table S2), which can inflate population cluster analyses and statistics (Wang 2018). After selecting a single representative for the identified duplicates, the final dataset contained 168,211 SNPs for 150 samples. The impact of duplicates on inferred population clusters in the network is demonstrated in Figure S1.

### 2.2 Hierarchical Network Analysis and Phylogeography

We used PLINK v1.9 (Chang et al. 2015) to calculate a pairwise identity‐by‐state (IBS) distance matrix, which corresponds to the complementary proportion of shared SNPs in a haploid organism, and is the recommended distance measure for *NetView* v1.0 (Neuditschko et al. 2012), the network analysis R package used here. NetView applies a combined k‐mutual nearest neighbor (k‐NN) network by plotting connections (i.e., edges) between individuals within each other's nearest neighbor group at resolution *k* and then assigns communities (i.e., cluster) using a community detection algorithm. We used the InfoMap algorithm for hierarchical population assignment since it is most sensitive to fine‐scale clusters in the data, aligning with the goal of our study, and the algorithm assigns clusters where random walks in the network consistently stay. Thus, exploring the network topology across different values of *k* and the changes in mutual nearest neighbors (NN) can provide broad‐ and fine‐scale patterns of population structure where a small *k* shows the strongest relationships at a fine‐scale and a large *k* includes more distant relationships at a broad‐scale. However, it should be noted that k‐NN is not a measure of algorithm accuracy and the network topology changes dramatically with resolution. Importantly, Neuditschko et al. (2012) recommend using the elbow method in the community detection plot to find the most likely k‐NN to describe the structure in the given dataset and exploring k‐NN for substructure depending upon the research question. We selected k‐NN values that plateau in the NetView *plotSelection* community detection graph, indicating locally stable cluster groups in the network and used the clusters for comparison to other popular structure methods (see Population Structure and Statistics).

The clusters inferred from the network were plot in geographic space using functions from R packages *popgraph* v1.6.0 (Dyer 2021), *ggplot2* v4.0.2 (Wickham 2016) and *ggsn* v0.5.3 (Santos Baquero 2019) to visualize relationships among samples across North and South America. Coordinates for each sample were used if published, otherwise they were estimated from the available metadata for visualization purposes.

### 2.3 Population Structure and Statistics

First, to compare the inferred broad‐scale populations from the network to other established methods, we used the parametric tool *ADMIXTURE* v1.3.0 (Alexander et al. 2009) with the –haploid flag, cross‐validation of 10, and the random seed set using the time. ADMIXTURE was run 30 times for each of 2–10 clusters (*K*), the number of assumed populations in the dataset. The most likely value of *K* was selected by the lowest cross‐validation score across replicates. The 30 admixture replicates were run through the online *CLUMPAK* (Kopelman et al. 2015) server to find the clustering consensus for each value of *K*.

Second, we validated the moderate‐scale population assignment by assessing the similarity to branching patterns in a phylogenetic tree. We ran IQTREE v 3.0.1 (Trifinopoulos et al. 2016), which identified 105,964 parsimony‐informative SNPs from the run parameters: GTR model, 1000 UltraFast bootstraps, and 0.4 perturbation strength. The tree converged after 2600 iterations and was plot in R using the package *phangorn* v3.0.0.0 (Schliep et al. 2017; Schliep 2011).

Lastly, we validated the fine‐scale population clusters by testing for patterns of genetic isolation using multiple population‐level statistics. The SNP data was transformed and converted in R using functions from *adegenet* v2.1.11 (Jombart 2008; Jombart and Ahmed 2011) and *hierfstat* v0.5‐11 (Goudet and Jombart 2022). Pairwise *F*_ST_ for haploid data was calculated among the network clusters using the *pairwise .WCfst* function following Weir and Cockerham's *F*_ST_ (Weir and Cockerham 1984), which includes a small‐sample size correction. *F*_ST_ confidence intervals were calculated from 5000 bootstraps using *boot.ppfst*. Pairwise *F*_ST_ and confidence intervals were plot using the R package *pheatmap* v1.0.13 (Kolde 2019). *F*_ST_ thresholds are interpreted where 0.05–0.15 indicates moderate differentiation, 0.15–0.25 high differentiation, and > 0.25 is very high differentiation (Wright 1965). Since more recent work has shown *F*_ST_ thresholds vary by organism and loci (Hall 2022; Nybom 2004), multiple measures of genetic diversity within each population are also reported. We also calculated within‐population gene diversity (*H*_*S*_) from *basic. stats* in the R package *hierfstat* to assess whether diversity within each population group was consistent. We used the function gl.report.pa from *dartR* v2.9.9.5 (Mijangos et al. 2022; Gruber et al. 2018) to identify private alleles within each population in comparison to the rest of clusters in the *same* species.

## 3 Results

### 3.1 Hierarchical Network Analysis and Phylogeography

The kinship analysis identified 11 groups of individuals that shared more than 99.9% of SNPs (Table S2), so a single representative was randomly selected for each group, reducing the dataset to 150 individuals and 168,211 SNPs. In total, 48 *C. immitis* and 102 *C. posadasii* whole‐genome sequences were retained. Among the retained isolates, genomic coverage with at least 5× sequencing depth ranged from 87.4% to 99.7% (Table S1).

**FIGURE 1:**
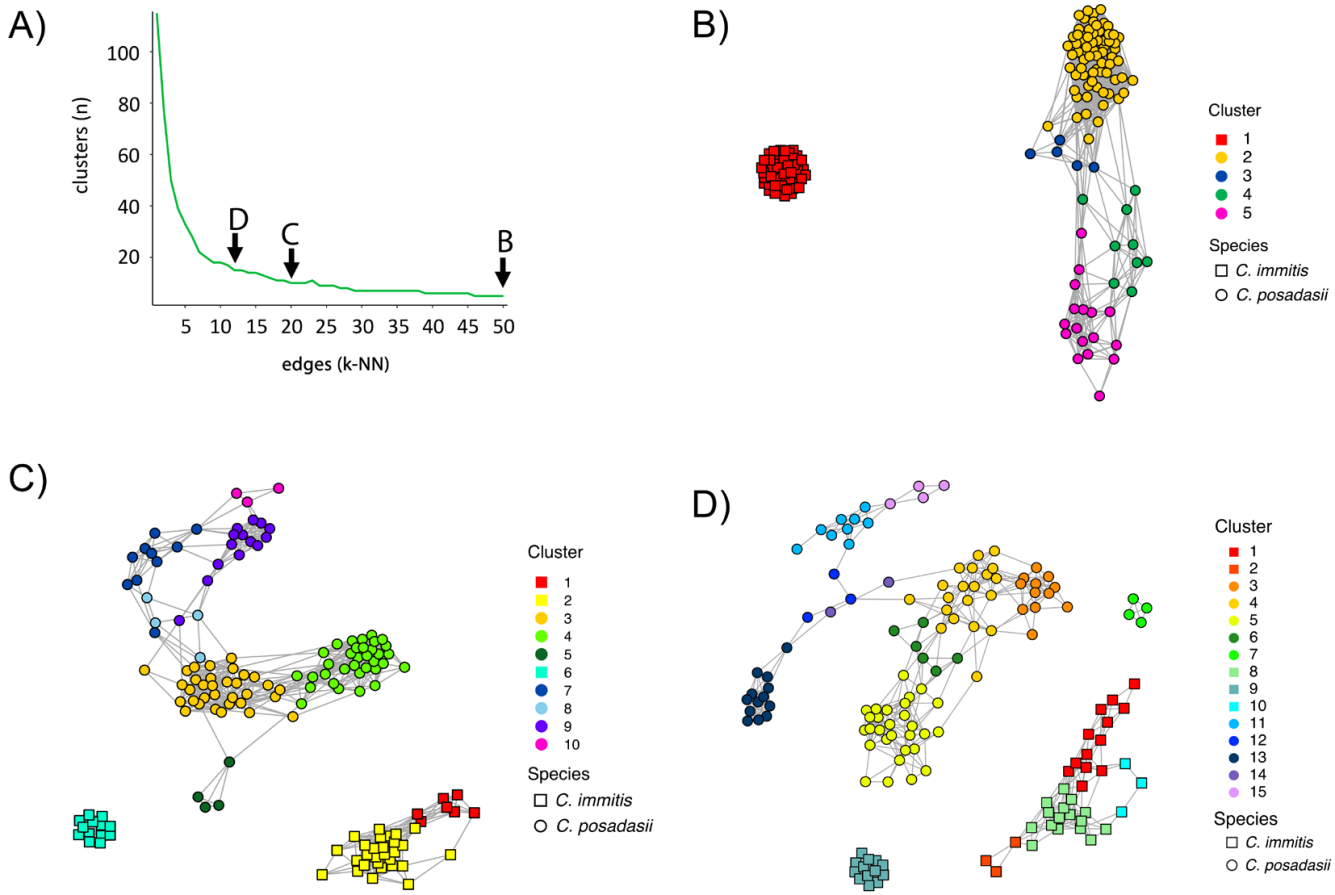
NetView network analysis plots for hierarchical population structure inference using publicly available whole‐genome sequence data for 102 *C. posadasii* and 48 *C. immitis* from NCBI. (A) Community detection plot showing the inferred clusters across sequential *k* mutual nearest neighbors (k‐NN) for the Infomap community detection algorithm. A small k‐NN limits the network to mutual neighbors that share the strongest relationships and equates to fine‐scale resolution, whereas a large k‐NN includes many more mutual nearest neighbors and includes weaker relationships at a broad‐scale resolution. Edges between nodes correspond to ranking in the k‐NN resolution where shorter edges correspond to higher rank and indicate closest neighbors, and longer edges correspond to lower rank or more distant neighbors. The two species are distinguished by their node shape where *C. posadasii* are circles and *C. immitis* are squares. (B) The broad‐scale network at k‐NN = 50 with 5 total clusters, (C) the moderate‐scale network at k‐NN = 20 with 10 total clusters and (D) the fine‐scale network at k‐NN = 12 with a total of 15 clusters.

We identified hierarchical broad‐ to fine‐scale population structure among and within each species by exploring the network across multiple k‐NN resolutions (Figure 1A). Starting with a k‐NN of 50, referred to as the broad‐scale network, 5 clusters were identified among the two species in the dataset with clusters associated with different broad‐scale geographic regions. *Coccidioides immitis* is represented by a single, dense cluster (Cluster 1, Figure 1B), and shares no edges with the *C. posadasii* network clusters. Four clusters were found among *C. posadasii*, consistent with expected network patterns for greater diversity and multiple ancestral lineages. Within *C. posadasii*, Clusters 2 and 5 are separated by smaller Clusters 3 and 4, suggesting the former are unique ancestral lineages and the latter are a mixture of the two (Figure 1B). Reducing k‐NN to 20, referred to as the moderate‐scale network, removes less informative edges allowing the network topography to open and show geographic substructure. At this resolution, *C. immitis* is represented by 3 clusters, one of which shares no edges with the rest of *C. immitis* (Cluster 3; Figure 1C), and 7 clusters were identified among *C. posadasii* with substructure apparent (Clusters 3 and 4: Figure 1C). Exploring k‐NN = 12, the fine‐scale network, the topography splits *C. immitis* into 5 clusters with Cluster 9 plot as an isolated group, which corresponds to the same individuals in Cluster 6 from the k‐NN = 20 network (Figure 1D and Table S1).

**FIGURE 2:**
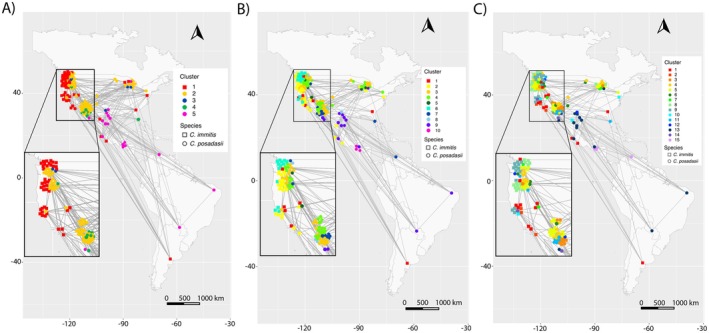
Plots showing the broad‐, moderate‐ and fine‐scale networks in geographic space for both *Coccidioides* species using reported locations for latitude and longitude points. (A) The broad‐scale network at k‐NN = 50, (B) the moderate‐scale network at k‐NN = 20, and (C) the fine‐scale network at k‐NN = 12. Squares denote *C. immitis* and circles denote *C. posadasii*. Edges in this plot are *not* proportional to k‐NN ranking and instead show mutual nearest neighbors at each network scale in relation to source location reported for each sample. Some geographic points are estimated based on available data for the samples and are only used for visualization purposes.

When viewing the networks in geographic space, patterns of geographic structure and likely travel‐affiliated infections become clear. At the broad scale, Cluster 5 is primarily comprised of cases reported in Texas, Mexico, and South America (TX/MX/SA) (Figure 2A). Cluster 2 is concentrated in Arizona but includes points in non‐endemic parts of the United States such as Michigan, Wisconsin, and Washington (Figure 2A). Isolates in Cluster 3 are from multiple different U.S. states including: Texas, Washington, Wisconsin and Michigan (Figure 2A). Cluster 4 is primarily associated with Tucson, Arizona, but was also reported in Florida, Oregon, and Sonora, Mexico (Figure 2A). These clusters show the broad‐scale patterns of population structure and distribution of ancestral lineages associated with broad geographic areas, although the isolates in non‐endemic regions can make it challenging to identify the geographic boundaries of those lineages. However, since isolates in non‐endemic regions are closely associated with ancestral lineages from known endemic areas, the network does not indicate establishment.

In the moderate‐scale network, multiple clusters appear to indicate substructure affiliated with smaller geographic regions. Among *C. immitis*, Cluster 6 contains 13 individuals that share no edges with the rest of the species that are primarily reported from Washington, two in the San Jaquin Valley of California, and a single individual from Oregon (Figure 2B). The isolation in the network suggests a unique genotype, likely isolated geographically with little gene flow from other populations. In contrast, Clusters 1 and 2 contain 7 and 28 individuals, respectively, and share edges between individuals from the opposite cluster indicating gene flow and range overlap (Figure 1C). These two clusters are reported across a broader geographic range including California, Washington, parts of Mexico, a single point in Argentina, and nonendemic regions in the United States (Figure 2B). Among *C. posadasii*, Clusters 3 and 4 contain 33 and 34 individuals, respectively. These two clusters split from Cluster 2 in the broad‐scale network (Figure 1C) and are primarily associated with Tucson (Cluster 3) and Phoenix (Cluster 4) (Figure 2B). Interestingly, Cluster 10 split from the broad‐scale TX/MX/SA cluster and contains the 3 individuals from Guatemala (Figure 2B). The grouping of Guatemala isolates suggests an introduction event from the broader TX/MX/SA group and establishment in that area. This indicates that after establishment, the Guatemala cluster has reduced gene flow with other clusters.

In the fine‐scale network, which corresponds to the strongest relationships between individuals in this dataset, clusters contain a range of 2–29 individuals with variable density in the network. *Coccidioides immitis* Clusters 1, 2, and 10 are reported from parts of northern and southern California and Mexico, whereas *C. immitis* Clusters 8 and 9 are predominantly reported in Washington and Oregon (Figure 2C). The samples in Cluster 9 are the same *C. immitis* samples from Cluster 6 in the moderate‐scale network. This cluster exhibits strong genomic isolation from the rest of *C. immitis* , indicative of little to no gene flow with other clusters. Cluster 8 plots in the middle of Clusters 1 and 2 in the network analysis with shared edges among the other clusters (Figure 1D), indicating a lack of genetic isolation and greater relatedness among these clusters. Due to the data preprocessing steps, only a single soil sample from Washington was included in the analysis, which grouped in Cluster 1 (Figure 2C). Interestingly, one of the nearest neighbors to the Washington soil sample is the isolate from Argentina. Among *C. posadasii*, Cluster 7 is isolated from the rest of the network (Figure 1D) and includes cases from Nevada, Washington, Michigan, and Wisconsin (Figure 2C). The Arizona samples split further into a Phoenix‐ (Cluster 5) and Tucson‐specific (Cluster 3) groups that are connected by intermediate Clusters 4 and 6 (Figure 2C).

### 3.2 ADMIXTURE

**FIGURE 3:**
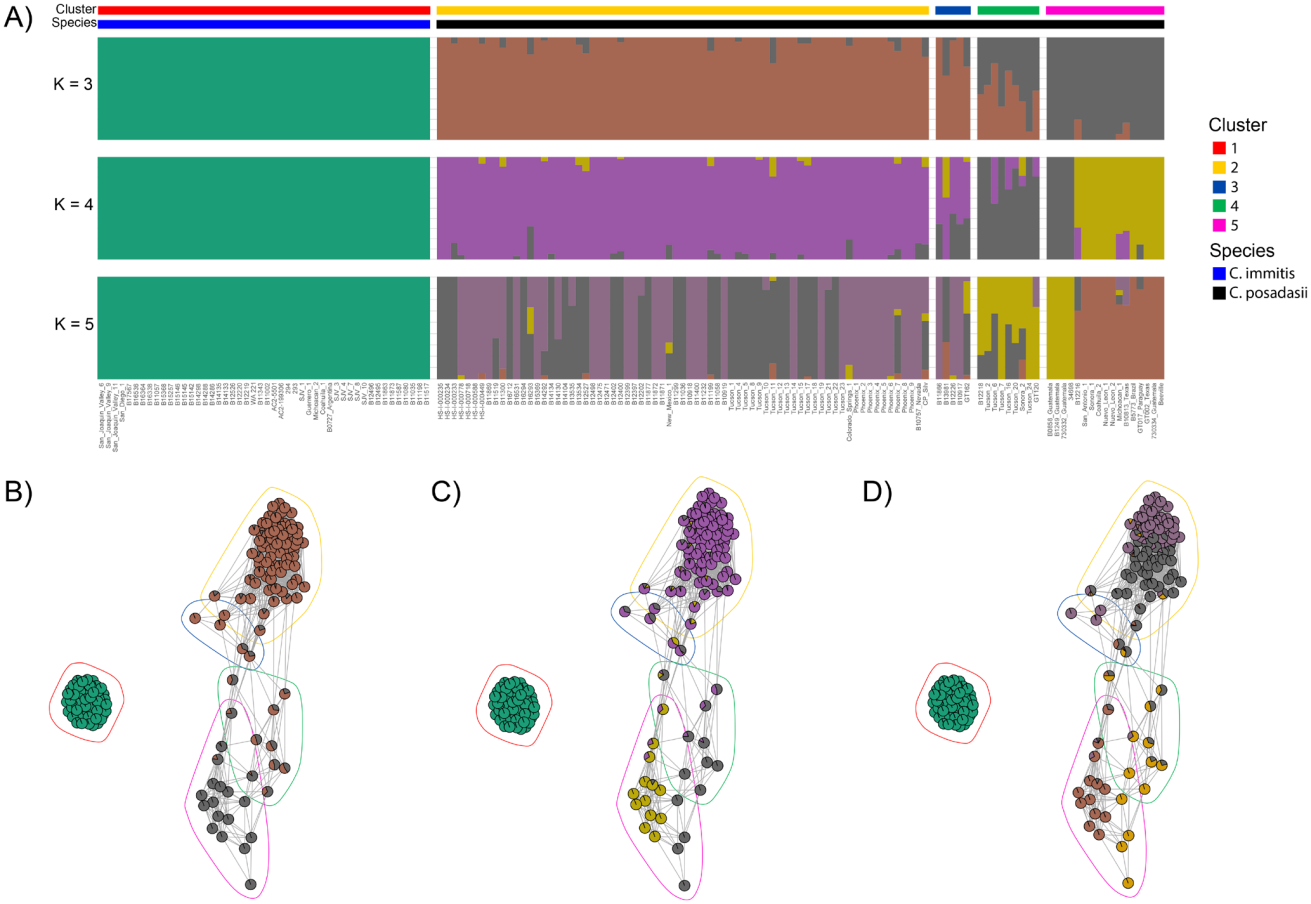
ADMIXTURE consensus barplots at *K* = 3–5 for both *Coccidioides* species and pie proportions combined with the broad‐scale network at each K. (A) The consensus barplots for *K* = 3–5 with *K* = 4 representing the optimal ancestral lineage estimate using the lowest cross validation score across 30 replicates. The cluster and species for each group in the barplots are shown in panels across the top where Clusters: 1 = red, 2 = yellow, 3 = blue, 4 = green, and 5 = pink and Species: blue = *C. immitis* and black = *C. posadasii*. The broad‐scale k‐NN = 50 network with nodes showing ADMIXTURE consensus proportions for each individual in the network at (B) *K* = 3, (C) *K* = 4, and (D) *K* = 5. Polygons in the networks circle the inferred Infomap clusters following the same Cluster color scheme. Edges in the network are proportional to the k‐NN ranking of mutual nearest neighbors where short edges correspond to higher rank and closer relationships, and long edges show lower rank and distant relationships.

The ADMIXTURE analysis inferred ancestral lineages consistent with the clusters identified in the broad‐scale network. The optimal number of ancestral populations (*K*) based on the lowest cross validation across 30 replicate runs was at *K* = 4 (Figure S2). *Coccidioides immitis* is estimated to have a single ancestral population regardless of *K*. At *K* = 3, ADMIXTURE identified 2 ancestral lineages among *C. posadasii* which correspond to Clusters 2 (Arizona) and 5 (TX/MX/SA) from the broad‐scale network (Figure 3A). The admixed samples were identified as two separate clusters in the network (Figure 1B; Clusters 3 and 4) that link the Arizona and TX/MX/SA lineages (Figure 3B). At *K* = 4, ADMIXTURE estimated 3 ancestral lineages among *C. posadasii* with Arizona still a single lineage; however, the Guatemala and Venezuela isolates are identified from the TX/MX/SA Cluster (Figure 3C). This supports the conclusion of historic introduction to that region and subsequent establishment and survival, but limited gene flow with other isolates. Lastly, at *K* = 5, ADMIXTURE identified two lineages among the Arizona samples, which are consistent with the Arizona clusters in the network (Figure 3D). Overall, the network output agrees with the ADMIXTURE analysis and indicates Arizona and TX/MX/SA are represented by diverged, ancestral lineages with substructure within each indicative of migration from each into surrounding geographic regions.

### 3.3 Phylogenetics

**FIGURE 4:**
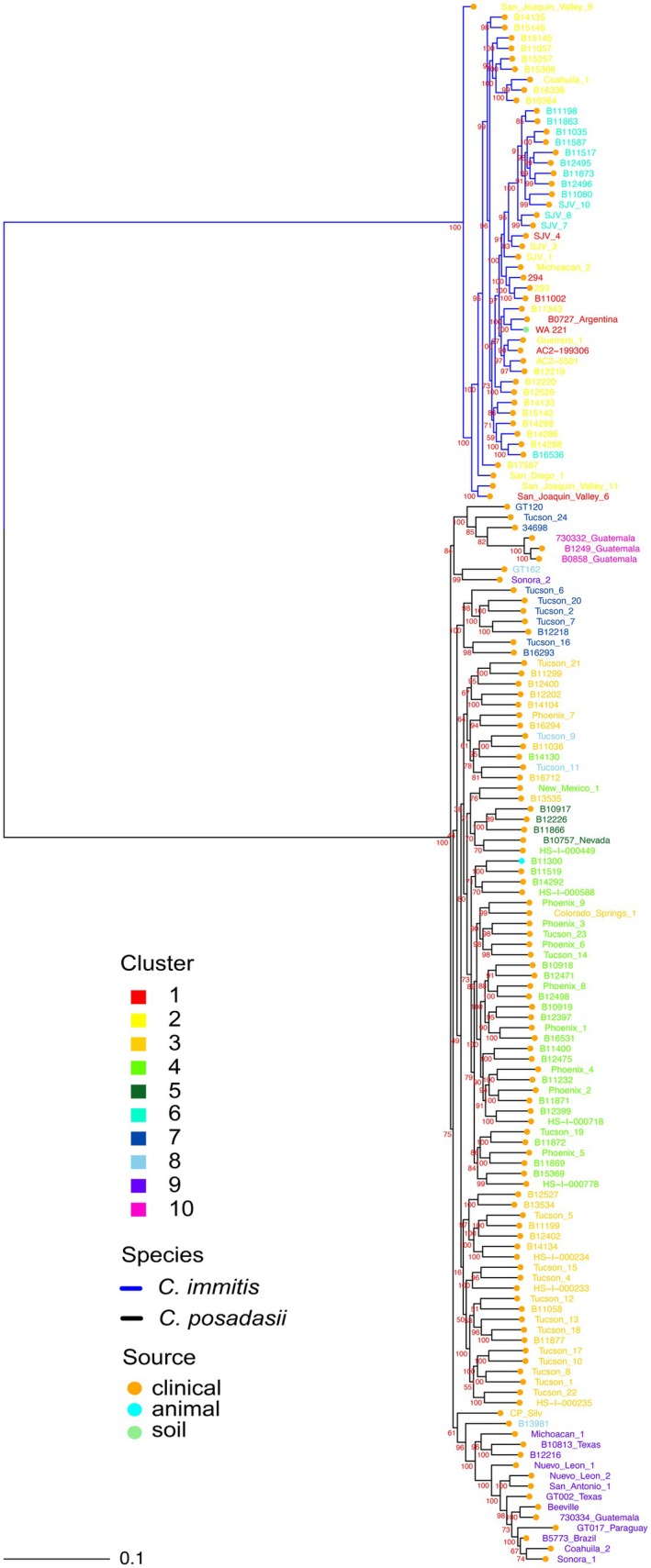
IQTREE phylogenetic tree for *Coccidioides posadasii* (*N* = 102) and *C. immitis* (*N* = 48). The tree branches are colored by species where *C. immitis* is blue and *C. posadasii* is black. The tree nodes are colored by isolation source with orange = clinical, green = soil, and cyan = animal. The tree labels are colored by the inferred clusters from the moderate‐scale k‐NN = 20 network for the 150 isolates. Bootstrap values are shown in red at each branch.

Comparing the phylogenetic tree to the clusters in the moderate‐scale network identified relatively consistent substructure among the two species; however, it performed poorly with the individuals exhibiting a mixture of multiple ancestral lineages. The tree placed Cluster 1 samples among Cluster 2 (Figure 4), which share edges between clusters in the network, suggesting Clusters 1 and 2 are more similar and exhibit gene flow. However, the structure identified by the network is not clear in the phylogenetic tree (Figure 4). In contrast, among *C. posadasii* the 7 network clusters are in relative agreement with the phylogenetic tree grouping (Figure 4). Cluster 10, corresponding to samples from Guatemala, shares a recent common ancestor with the sample from Venezuela; however, at this resolution in the network, the sample from Venezuela is clustered with the TX/MX/SA group in Cluster 7 (Figure 4). The branch length for the Guatemala samples suggests it belongs to a unique clade, consistent with the clustering in the network. However, the network identified Cluster 5 as a strong cluster group, indicated by the short edges between cluster members but long edges connecting to Cluster 4 (Figure 1C). In the phylogenetic tree, the samples in Cluster 5 branch from a recent common ancestor nested among Clusters 3 (Tucson) and 4 (Phoenix) but would not otherwise be distinguishable as a unique genotype. Samples from Clusters 3 and 4 are grouped consistently in the tree (Figure 4) and reflect the shared edges between the two clusters from the network (Figure 1C). Although the two methods seem to be in relative agreement on sample placement, the bifurcating nature of the phylogenetic tree does not seem to represent complex evolutionary relationships well.

### 3.4 Population Statistics

**FIGURE 5:**
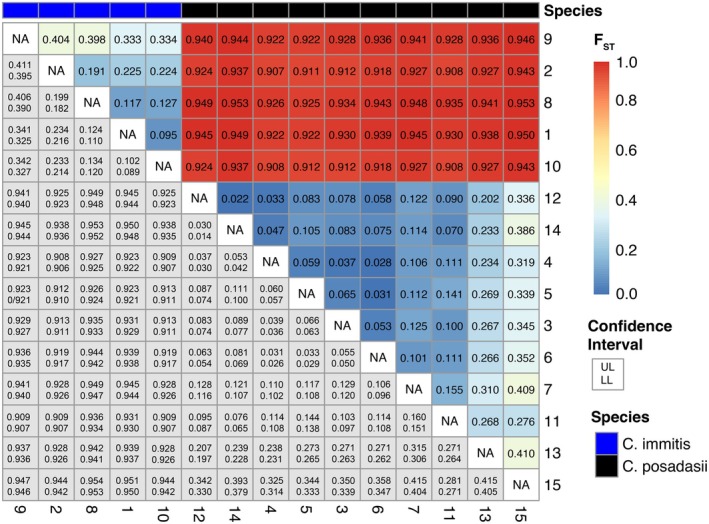
Pairwise *F*_ST_ heatmap and bootstrap confidence intervals comparing population differentiation between the inferred *Coccidioides* clusters from the fine‐scale k‐NN = 12 network. The upper triangle shows the pairwise *F*_ST_ values and the lower triangle shows the confidence interval from 5000 bootstraps where the top value notes the upper limit, and the bottom value shows the lower limit. The species are noted in the panel at the top of the heatmap with *C. immitis* as blue and *C. posadasii* as black.

The pairwise *F*_ST_ and bootstrap confidence intervals indicate moderate‐ to high‐differentiation among the fine‐scale clusters. It should be noted that Clusters 2, 6, 7, 10, 12, 14 and 15 have small sizes and should be interpreted with caution (Table 1), but we attempted to mitigate small sample size effects by using Weir and Cockerham's (1984) *F*_ST_. The large SNP dataset also helps compensate for the effects of small populations (Willing et al. 2012), and the bootstrap upper and lower limits are included to show the variation among estimates. The two species are highly differentiated regardless of cluster comparison *F*_ST_ > 0.9 (Figure 5). The *Coccidioides immitis* Clusters 1 and 10 are most similar among the clusters, *F*_ST_ = 0.095, 95% CI [0.102, 0.089] (Figure 5). Interestingly, the three individuals in Cluster 10 connect California samples between Cluster 1 and 8; however, Clusters 1 and 8 are moderately differentiated *F*_ST_ = 0.117, 95% CI [0.124, 0.110] (Figure 5). Fascinatingly, Cluster 8 is nearly completely reported in Oregon and Washington; however, 4 of the cases reported travel to Mexico and California. Cluster 9 exhibits the highest differentiation among the *C. immitis* clusters (*F*_ST_ = 0.333–0.404) and lowest genetic diversity (Table 1 and Table S2), providing support for the cluster's isolation in the network. The remaining *C. immitis* groups are moderate to highly differentiated *F*_ST_ = 0.117–0.225 (Figure 5) with a range of private alleles in each cluster (Table 1). Among *C. posadasii*, Clusters 3, 4, 5 and 6 are all associated with parts of Arizona (AZ) and exhibit the lowest differentiation among the *C. posadasii* clusters *F*_ST_ = 0.031–0.065 (Figure 5). Clusters 4 and 6 are intermediate to Phoenix and Tucson in the network with the lowest differentiation between the clusters *F*_ST_ = 0.028, 95% CI [0.026, 0.031] (Figure 5). The Guatemala and Venezuela group (Cluster 15) exhibits the highest differentiation among the *C. posadasii* clusters, *F*_ST_ = 0.276–0.410 (Figure 5). The percent of missing SNPs in each population is less than 3%, with the majority under 1% (Table 1), indicating the missing loci are not contributing to the population structure. Gene diversity within each population is consistent across populations of each species with *H*_*S*_ ranging from 0.036 to 0.054 for *C. posadasii* and 0.024–0.029 for *C. immitis* (Table 1).

**TABLE 1:** Within population metrics and statistics for the 15 clusters identified in the fine‐scale k‐NN = 12 network.

Cluster	Species	Number of individuals	Total alleles	Private alleles	Percent missing alleles	*H*_S_
1	*C. immitis*	13	181,544	189	2.12%	0.0259
2	*C. immitis*	3	172,998	107	1.32%	0.0266
3	*C. posadasii*	12	195,573	86	0.01%	0.0531
4	*C. posadasii*	20	204,983	242	0.49%	0.0541
5	*C. posadasii*	29	206,139	271	0.57%	0.051
6	*C. posadasii*	6	187,688	53	0.60%	0.0526
7	*C. posadasii*	4	181,706	39	0.60%	0.0493
8	*C. immitis*	16	181,072	1	0.60%	0.0242
9	*C. immitis*	13	184,414	3	1.12%	0.0295
10	*C. immitis*	3	173,115	0	2.95%	0.0266
11	*C. posadasii*	9	193,175	181	0.62%	0.0573
12	*C. posadasii*	3	180,892	36	0.77%	0.0571
13	*C. posadasii*	13	189,643	238	1.11%	0.0432
14	*C. posadasii*	2	175,115	42	0.23%	0.0503
15	*C. posadasii*	4	177,653	73	1.18%	0.0356

*Note:* H_S_: within population gene diversity as defined by the function *basic. stats* in *hierfstat*.

## 4 Discussion

We demonstrated the utility of a genomic network analysis in identifying geographic structure and inferring likely infection locations in *Coccidioides* spp., the causative agent of Valley fever, which produced comparable results to a combination of traditional methods. Using a kinship analysis to reduce genetic duplicates followed by a hierarchical network and structure assessments helped elucidate these patterns. Our comparison grouped samples into consistent clusters at the broad‐ and moderate‐scales but also identified fine‐scale structure and travel‐associated cases not reported thus far.

Many samples in the dataset were reported outside the known endemic range for the two species, but the network suggests those infections were acquired from travel to endemic regions (Figure 1D). Importantly, the isolates reported in non‐endemic regions are not represented by a unique genetic cluster or strong fragmentation in the network, which would be expected if local establishment and spread occurred. In contrast, the isolated Cluster 9 (Figure 1D) among *C. immitis* contained samples primarily reported in Washington and Oregon; however, previous publications and travel history anchor the genotype to California and contain isolates from the San Joaquin Valley (Monroy‐Nieto et al. 2023; Engelthaler et al. 2016). This indicates infection most likely happened during travel to California, and diagnosis occurred in Washington or Oregon. Additionally, the individuals in Cluster 9 remain isolated in the network across multiple k‐NN and do not share nearest neighbors with the Washington soil isolate, so establishment in Washington seems unlikely. Instead, the Washington soil representative clusters closely with a historic clinical isolate from Argentina (Figure 1D), which suggests the Argentina infection was likely from Washington and matches findings by Engelthaler et al. (2016). Since *C. posadasii* is the only reported species in Argentina‐acquired clinical infections (Viale et al. 2024; Canteros et al. 2010), it seems unlikely this *C. immitis* infection occurred in Argentina. In the absence of complete metadata, inferring the infection source would be challenging and misleading if relying on metadata alone. Similar inferences of travel‐associated infections from parts of Arizona (Clusters 3 and 5; Figure 2D) are supported across broad geographic locations in the United States which, when combined with other measures of population structure, provide a robust methodology for studying patterns of infection in emerging diseases.

Additionally, we detected patterns of finer‐scale populations among small geographic regions in the southwest United States that have not been explored thus far (Figure 2C). In the moderate‐scale network, multiple samples from Tucson (*n* = 6) share mutual nearest neighbors with isolates from Venezuela and Guatemala (Figure 1C), and form two separate clades in the phylogenetic tree (Figure 4). Those same isolates in the fine‐scale network split into two separate clusters with the Venezuela and Guatemala isolates forming the majority of Cluster 15 (Figure 1D) and five of the Tucson isolates forming the majority of Cluster 11 (Figure 1D). While the Tucson samples have formed small clades in previous studies (Mead et al. 2022), their placement is not always with the Caribbean group (Monroy‐Nieto et al. 2023; Teixiera et al. 2019; Engelthaler et al. 2016); however, they are consistently plot together and have been referred to as Tucson2 by Monroy‐Nieto et al. (2023). It is unclear if the Tucson samples are an Arizona or a Caribbean subpopulation since they share common ancestry with the Venezuela/Guatemala isolates at K = 4 (Figure 3A; Cluster 4) but exhibit high differentiation from the four Venezuela/Guatemala isolates in the fine‐scale network. While the small sample size makes conclusions challenging, the persistence of this structure across our analyses and other papers indicates the pattern is not an artifact. Similarly, Cluster 7 in the fine‐scale network is isolated from the rest of *C. posadasii* and contains the isolate B10757_Nevada, which might indicate a Nevada or northern Arizona subpopulation since two of the cluster members have confirmed travel to Arizona (Monroy‐Nieto et al. 2023). While Cluster 7 only contains four samples, three of the four share the highest pairwise kinship metric (> 0.95) with each other and the fourth sample, however the fourth sample shares high kinship metrics with other samples from Arizona (Table S2). While this is also a small sample size, the cluster forms a clade in the phylogenetic tree and remains isolated from the rest of *C. posadasii* across a range of k‐NN, indicating stable structure within these data that is similar to clade structure in Monroy‐Nieto et al. (2023). These results provide fascinating patterns to follow up on in future studies to assess the validity of substructure in Nevada, parts of Arizona, and the Caribbean, and elucidate what might be contributing to the fine‐scale patterns. The consistency in this study to traditional methods as well as other studies demonstrates the applicability of a genomic network analysis in exploring population structure and inferring most likely source location of travel associated cases in this disease system.

The network identified population structure across hierarchical levels that is consistent with the literature and demonstrates the method's applicability in identify structure in complex disease systems. As anticipated, *C*. *posadasii* and *C. immitis* exhibit strong species division and substructure identified at multiple geographic scales, for example, hierarchical levels. Depending on the markers used and number of samples, previous studies have identified similar broad‐scale patterns of structure among the two species with *C. immitis* represented by a single ancestral lineage and *C. posadasii* represented by multiple (Viale et al. 2024; Mead et al. 2022; Teixiera et al. 2019; Alvarado et al. 2018; Luna‐Isaac et al. 2014), with the exception of Teixeira and Barker (2016) who identified two lineages among *C. immitis* in California. *Coccidioides immitis* is estimated to have diverged from *C. posadasii* 300 thousand years ago whereas *C. posadasii* shows evidence of being much older and more diverse (Engelthaler et al. 2016; Neafsey et al. 2010), which is reflected in a single ancestral lineage for *C. immitis* and more for *C. posadasii* (Figure 3A). The populations identified among *C. posadasii* corresponded to groups inferred in previous studies, notably the TX/MX/SA cluster with a Caribbean subgroup containing Guatemala and Venezuela samples and a large Arizona group with substructure in Phoenix and Tucson (Monroy‐Nieto et al. 2023; Mead et al. 2022; Teixiera et al. 2019; Engelthaler et al. 2016). Broad‐ to fine‐scale structure in these species, indicated by moderate‐ to high‐ differentiation, suggests limited to no gene flow among subpopulations in regions of California and Arizona. Interestingly, the network also placed admixed individuals into clusters intermediate to the non‐admixed clusters, which is more intuitive than the phylogenetic tree. This appears to be an example of how phylogenetic trees have trouble with admixed genotypes (Feng et al. 2022) and highlights how a network can better visualize such patterns across hierarchical scales in a single analysis.

The fine‐scale statistics suggest different degrees of isolation among the populations identified here, which requires additional ecological work to better understand what is limiting gene flow. Although we did not test for isolation‐by‐distance (IBD) since some latitude/longitude points were estimated and many cases were reported outside the endemic area, there is a pattern of geographic‐genotype association, which is well documented among a variety of species (Aghbolaghi et al. 2023; Bataille et al. 2011; Suni and Gordon 2010). The city‐specific genotypes among Arizona isolates and multiple genotypes present in California indicate that long‐distance dispersal and subsequent gene flow are limited. While wind is a dispersal mechanism in *Coccidioides* spp. (Porter et al. 2024; Gade et al. 2020) and is linked to infection events depending on how dust events are defined (Tong et al. 2017, 2022; Comrie 2021), the range of wind dispersal is still unknown. However, since the present dataset is primarily clinical isolates, dispersal among environmental isolates might find that particular genotypes are better suited to long‐distance dispersal than others, as was found in the plant pathogen *Epichloë* spp. (Treindl et al. 2023). It is imperative to include more environmental data in future studies since this pattern of genetic differentiation contrasts with other pathogenic fungal species that exhibit frequent recombination and extensive gene flow (Mei et al. 2020). Even with the lack of environmental data, these method comparisons demonstrate the validity of the network in grouping samples into biologically relevant populations and improve our ability to visualize complex evolutionary processes from a hierarchical perspective.

Although the network analysis provided biologically relevant output, distance‐based clustering methods have limitations and should still be paired with other methods for validation. While NetView is easy to implement, clusters in a network are dependent upon distance metric and clustering algorithm used (Pritchard et al. 2000) and requires thorough understanding of what tool is best suited to the data. In our analysis, the network grouped admixed individuals intermediate to their dominant ancestral lineage; however, the clusters each admixed individual was placed into changed with resolution and mutual nearest neighbors. Comparing to the literature, the same samples have been placed in different clades across studies, but no two publications used the exact same datasets (Mead et al. 2022; Teixiera et al. 2019; Engelthaler et al. 2016). It seems that these admixed individuals are most likely from an area of geographic range overlap and additional environmental samples are needed to better understand the range of each cluster, anchor clinical genotypes in geographic space as in Teixeira and Barker (2016). While distance‐based networks have limitations, methods improvements have been published (Greenbaum et al. 2016) and provide a useful tool with no model assumptions or need for a priori population assignment.

The endemic region for *Coccidioides* spp. is projected to expand under different climate change scenarios (Gorris et al. 2019), so monitoring population structure and patterns of infection will be paramount to mapping range expansion in the future. Using an unsupervised machine learning method to group individuals into populations and infer patterns of infection can improve our biosurveillance capabilities. Machine learning methods improve upon model‐based tools by limiting a priori assumptions and making predictions and inferences from patterns inherent to the data (Huang et al. 2024; Korfmann et al. 2023). Deep learning has been successful at predicting disease transmission risk factors and outcomes in a variety of pathogens (Pillai et al. 2022). These tools have the potential to improve disease outbreak monitoring by reducing the computational needs to work with big datasets and harness the genomic information of samples with unknown source or infection location. Future work can incorporate more complex ML methods to identify genotypes associated with severe disease, environmental adaptations, and other phenotypic traits relevant to emerging pathogens. However, the need for training datasets in predictive ML greatly reduces the pathogens that can be used (Kawasaki et al. 2025). Small datasets with few individuals or few genomic variants are not good candidates for ML applications since the value of ML is that it can handle large, complex data efficiently. Future applications with predictive goals will also need to be hallucination‐aware and confirm the interpretability of the output (Al Meslamani et al. 2024).

Our study provides a promising example of how an unsupervised ML‐based genomic network analysis can improve our understanding of pathogen ecology, group cases into populations and infer travel‐associated infections at multiple geographic scales. Identifying pathogen populations can provide a valuable basis from which to infer genetic variants conducive to severe disease, at‐risk populations, or adaptations specific to local environments. As new pathogens emerge and big data becomes more common, ML has the potential to improve our ability to combine high‐dimensional genomic data with multivariate phenotype and environmental characteristics for a better understanding of pathogen ecology and transmission dynamics.

## Author Contributions

**Cari D. Lewis:** conceptualization (equal), formal analysis (lead), methodology (lead), visualization (lead), writing – original draft (lead). **Morgan E. Gorris:** conceptualization (equal), funding acquisition (equal), supervision (equal), writing – review and editing (equal). **Kimberly A. Kaufeld:** conceptualization (equal), funding acquisition (equal), supervision (equal), writing – review and editing (equal). **Andrew W. Bartlow:** conceptualization (equal), funding acquisition (equal), supervision (equal), writing – original draft (supporting), writing – review and editing (equal).

## Funding

Funded by a Department of Energy Laboratory Directed Research and Development grant (Number: YW0M00) through Los Alamos National Laboratory, an affirmative action/equal opportunity employer, managed by Triad National Security LLC, for the National Nuclear Security Administration of the U.S. Department of Energy under contract 89233218CNA000001.

## Conflicts of Interest

The authors declare no conflicts of interest.

## Supporting information


**Figure S1:** Network output for similar k‐NN values to demonstrate the effect of genetic duplicates on inferred clusters. (A) Network and community detection plot for k‐NN = 11 for 183 individuals. The red polygons show the clusters with genetic duplicates. (B) The deduplicated network with 150 individuals at k‐NN = 12. The nodes are colored by the assigned cluster from the network from (A).
**Figure S2:**. Cross‐validation (CV) plot from 30 ADMIXTURE replicates (gray) and the average across runs (blue). The lowest CV across runs is at *K* = 4.

**Table S1:** Metadata, alignment and deduplication metrics, and hierarchical cluster assignment for the 183 and reduced dataset of 150 *Coccidioides* spp. used in the network analysis and population structure assessments.
**Table S2:** The pairwise kinship analysis showing proportion of shared SNPs within and among the two *Coccidioides* species. Pairs with > 0.999 shared SNPs are highlighted in red and show groups that were reduced to a single representative.

## Data Availability

The analyses conducted in this manuscript were done using publicly available data with all relevant information in the manuscript. No new data was generated in the preparation of this manuscript.
