# Supplementary material for: Assessing the Application of a Genomic Network Analysis in Population Ecology: Inferring Patterns of Dispersal and Geographic Structure in the Emerging Pathogen, Coccidioides
Source: Ecol Evol. 2026 Apr 7;16(4):e73452. doi: 10.1002/ece3.73452 (PMC13054236; doi:10.1002/ece3.73452)
Supplement: Supplementary file 1 — Figure S1: Network output for similar k‐NN values to demonstrate the effect of genetic duplicates on inferred clusters. (A) Network and community detection plot for k‐NN = 11 for 183 individuals. The red polygons show the clusters with genetic duplicates. (B) The deduplicated network with 150 individuals at k‐NN = 12. The nodes are colored by the assigned cluster from the network from (A). Figure S2:. Cross‐validation (CV) plot from 30 ADMIXTURE replicates (gray) and the average across runs (blue). The lowest CV across runs is at K = 4. [file ECE3-16-e73452-s001.docx]

**Supplemental Figure 1**. Network output for similar k-NN values to demonstrate the effect of genetic duplicates on inferred clusters. A) Network and community detection plot for k-NN=11 for 183 individuals. The red polygons show the clusters with genetic duplicates. B) The deduplicated network with 150 individuals at k-NN=12. The nodes are colored by the assigned cluster from the network from A.

**Supplemental Figure 2**. Cross-validation (CV) plot from 30 ADMIXTURE replicates (gray) and the average across runs (blue). The lowest CV across runs is at K=4.

**Supplemental Table 1**. Metadata, alignment and deduplication metrics, and hierarchical cluster assignment for the 183 and reduced dataset of 150 *Coccidioides* spp. used in the network analysis and population structure assessments.

**Supplemental Table 2**. The pairwise kinship analysis showing proportion of shared SNPs within and among the two *Coccidioides* species. Pairs with > 0.999 shared SNPs are highlighted in red and show groups that were reduced to a single representative.
